# A Multi-Strategy Enhanced Whale Optimization Algorithm for Long Short-Term Memory—Application to Short-Term Power Load Forecasting for Microgrid Buildings

**DOI:** 10.3390/s26031003

**Published:** 2026-02-03

**Authors:** Lili Qu, Qingfang Teng, Hao Mai, Jing Chen

**Affiliations:** School of Automation and Electrical Engineering, Lanzhou Jiaotong University, Lanzhou 730070, China

**Keywords:** CEEMD (Complementary Ensemble Empirical Mode Decomposition), forecasting, microgrid, optimization, power load

## Abstract

High-accuracy short-term electric load forecasting is essential for ensuring the security of power systems and enhancing energy efficiency. Power load sequences are characterized by strong randomness, non-stationarity, and nonlinearity over time. To improve the precision and efficiency of short-term load forecasting in microgrids, a hybrid predictive model combining Complementary Ensemble Empirical Mode Decomposition (CEEMD) and a multi-strategy enhanced Whale Optimization Algorithm (WOA) with Long Short-Term Memory (LSTM) neural networks has been proposed. Initially, this study employs CEEMD to decompose the short-term electric load time series. Subsequently, a multi-strategy enhanced WOA with chaotic initialization and reverse learning is introduced to enhance the search capability of model parameters and avoid entrapment in local optima. Finally, considering the distinct characteristics of each component, the multi-strategy improved WOA is utilized to optimize the LSTM model, establishing individual predictive models for each component, and the predictions are then aggregated. The proposed method’s forecasting accuracy has been validated through multiple case studies using the UC San Diego microgrid data, demonstrating its reliability and providing a solid foundation for microgrid system planning and stable operation.

## 1. Introduction

Power load forecasting is of great significance for the planning and operation of power systems. Depending on the forecasting time, power load forecasting can be classified into very-short-term load forecasting (VSTLF), short-term load forecasting (STLF), medium-term load forecasting (MTLF), and long-term load forecasting (LTLF). Short-term load forecasting is typically used for the daily operation of power systems, such as the scheduling of power generation and transmission. With the development of smart grids, the importance of short-term load forecasting is increasing day by day [1,2]. Moreover, to mitigate the impact of flexible load uncertainties on power grid scheduling and to improve the accuracy of short-term load forecasting, it has become increasingly important. Precise and rapid load forecasting for microgrids actively contributes to the rational formulation of operational dispatch plans, facilitating the efficient, reliable, stable, and secure operation of microgrids in conjunction with the main power grid [3].

The usage functions of microgrid buildings determine that their loads have obvious periodicity. For example, teaching buildings consume a large amount of electricity during teaching periods and have a low load at night; student service centers have a high load during the day when students are active and a reduced load at night. However, in addition to the regular periodic changes, there are also many influencing random factors. Sudden equipment failures and special events can all cause unpredictable fluctuations in the load. At the same time, for example, police buildings need to keep equipment running throughout the day, and the load is relatively stable even during rest periods, while music buildings experience a sharp increase in load when there are performances or activities due to the concentrated use of equipment, and the load is low at ordinary times. These load differences among different building types increase the complexity and diversity of load data. Therefore, the load differences make the data exhibit a high degree of complexity and non-linearity, requiring the prediction model to have stronger adaptability and generalization ability to accurately capture the laws of load changes.

The remainder of this paper is arranged as follows. In Section 2, we discuss the related work. In Section 3 and Section 4, we introduce the methodology of the proposed model. In Section 5, we present the experimental results and analysis. Finally, in Section 6, we conclude the paper and provide future work directions.

## 2. General Introduction to STLF of Microgrid Load Forecasting

The power load in microgrids exhibits randomness and volatility, making it challenging to extract features from raw data. Consequently, many studies employ time-series decomposition methods, such as wavelet decomposition, empirical mode decomposition (EMD), and variational mode decomposition. A method using EMD to decompose the power load time series sequentially was proposed in [4], and long short-term memory (LSTM) networks were employed to predict electricity consumption for the next 24 h. However, EMD suffers from the issue of mode mixing. Ensemble empirical mode decomposition (EEMD), which adds white noise to EMD, mitigates the mode mixing problem to some extent. Yuan et al. used EEMD to decompose the load sequence into several regular intrinsic mode functions (IMFs) to reduce errors caused by the sequence’s randomness [5]. Nevertheless, the white noise cannot be completely canceled out, leaving residuals that introduce noise in the reconstructed signal. Complementary ensemble empirical mode decomposition (CEEMD) was thus introduced to counteract the white noise after reconstruction. Shi et al. utilized CEEMD to reduce the complexity of load data samples [6]. In light of the random and complex nature of short-term load in microgrids, this study employs CEEMD for decomposition and feature extraction from the raw signal. Comparative analysis reveals that this approach effectively enhances the accuracy of load forecasting.

Effective predictive models can enhance the accuracy of electric load forecasting to a certain extent. In ref. [7], a short-term electric load forecasting method based on machine learning techniques was proposed, where the XGBoost algorithm demonstrated superior performance, surpassing historical weekly predictions based on neural networks. Study [8] introduced a short-term electric load forecasting method that integrates various machine learning models, including XGBoost, Support Vector Machine (SVM), and Extreme Learning Machine (ELM), and employed an optimization algorithm to select hyperparameters for ELM, thereby improving the forecasting accuracy compared to traditional machine learning models. Machine learning has exhibited limitations in processing time-series data. Leveraging the strengths of time-series processing, models such as LSTM networks have begun to be utilized and have shown promising results. In ref. [9], a short-term load forecasting method based on LSTM that incorporates a feature attention-based encoder was proposed. Study [10] presented a hybrid Prophet-LSTM model that overcomes the slow convergence issue inherent in traditional LSTM models, offering smaller errors and computational times. Concurrently, more advanced deep learning models have been employed in load forecasting. Sara Atef et al. proposed deep-stacked unidirectional (Uni-LSTM) and bidirectional (Bi-LSTM) networks, with the optimized BiLSTM model demonstrating better performance in terms of Root Mean Square Error (RMSE) and Mean Absolute Percentage Error (MAPE) [11]. In ref. [12], an attention-based convolutional neural network (CNN) combined with LSTM and bidirectional BiLSTM was proposed. This method leverages CNN for feature extraction and combines LSTM with BiLSTM to predict the load for the next hour, outperforming individual CNN-BiLSTM and CNN-LSTM models.

Deep learning models are extensively utilized; however, the performance of these models is significantly influenced by multiple hyperparameters, with optimal hyperparameters being crucial for the models to achieve their full potential. In ref. [13], a genetic wind-driven (GWDO) optimization algorithm was proposed for fine-tuning the hyperparameters of factored conditional restricted Boltzmann machines (FCRBMs). Comparative analysis with existing models demonstrated that the optimization of hyperparameters significantly enhanced the model’s performance. Dai et al. introduced a second-order oscillation and repulsion particle swarm optimization algorithm for optimizing the hyperparameters of ensemble models [14], while Ehab et al. proposed a modified grasshopper optimization algorithm (MGOA) for hyperparameter selection. Results indicated that the employment of advanced optimization algorithms can effectively improve the accuracy of short-term electric load forecasting [15]. However, traditional optimization algorithms suffer from slow convergence and a propensity to fall into local optima. Therefore, this study proposes a multi-strategy improved whale optimization algorithm (MIWOA). Initially, this research employs Tent chaotic mapping for population initialization. The Tent chaotic map is adopted to replace the original population initialization method; it can generate sequences with high randomness and uniform distribution, which not only enhances the diversity of the population but also provides the algorithm with better global search capabilities. Subsequently, Sinusoidal Chaotic Mapping is utilized to increase the randomness of parameters within the WOA, preventing entrapment in local optima. Finally, to further improve convergence speed and precision, the Lagrange interpolation method is employed for position updates. Comparative tests of the proposed method on benchmark functions from CEC 2005 demonstrate that MIWOA can effectively enhance convergence speed and precision, rapidly obtaining optimal values and achieving rapid and accurate optimization of key parameters in the LSTM model.

In summary, to extract the features of the microgrid load time series and to mitigate the interference of irrelevant information, this paper applies the CEEMD to decompose the original power time series and proposes a multi-strategy improved WOA to optimize the parameter selection of the LSTM model, introducing the CEEMD-MIWOA-LSTM combined forecasting model. To validate the excellence of the proposed algorithm, comparisons are made using the CEC 2005 benchmark test functions, confirming its superiority. Finally, the MIWOA is utilized to search for the optimal key learning parameters of the LSTM model, which are then verified through multiple instances on the UC San Diego microgrid data and compared with other forecasting methods. The results demonstrate that the proposed model achieves the best predictive accuracy. Overall, this paper makes the following contributions:

1. A hybrid prediction model based on CEEMD and MIWOA-optimized LSTM is proposed for short-term power load forecasting in micro-grids.
2. The original power load time series is decomposed and feature-extracted by the CEEMD method, effectively improving the accuracy of load forecasting.
3. The MIWOA is introduced to optimize the key parameters of the LSTM model, significantly enhancing the model’s convergence speed and optimization accuracy.
4. Through four practical case studies, the applicability and accuracy of the proposed model in different building types are verified. High *R*^2^ values are obtained, demonstrating the superior performance of the model.

## 3. Power Load Decomposition Based on CEEMD

### 3.1. Empirical Mode Decomposition

The Empirical Mode Decomposition method, proposed by Huang et al., was initially developed for the analysis of nonlinear and non-stationary time series [16]. This approach decomposes a time-domain signal into a set of Intrinsic Mode Functions (IMFs) based on the signal’s own local characteristic time scales. For a given signal *x*(*t*), the EMD algorithm breaks it down into multiple IMF components that satisfy the following conditions: (1) the number of extrema and the number of zero crossings of an IMF must be equal or differ by at most one throughout the entire time span; (2) the mean value of the envelopes defined by the local maxima and local minima at any time point should be zero. The specific steps of the EMD are as follows:

(1) Identify all the maxima and minima of the signal and fit the upper envelope $h_{1}\left(t\right)$ and the lower envelope $h_{2}\left(t\right)$ using cubic spline interpolation, respectively. Calculate the mean $m_{1}\left(t\right)$ of the upper and lower envelopes, which is given by
  (1) $$m_{1}(t)=0.5\left[h_{1}(t)+h_{2}(t)\right]$$
(2) Compute the difference between *x*(*t*) and $m_{1}\left(t\right)$ to obtain the $u_{1}\left(t\right)$ component, which is expressed as
  (2) $$u_{1}(t)=x(t)-m_{1}(t)$$
(3) Using the aforementioned criteria for IMF selection to determine the sequence $u_{1}\left(t\right)$, if it does not meet the conditions, it cannot be considered an IMF. Processes (1) and (2) are then repeated until the sequence $u_{k}\left(t\right)$ satisfies the conditions for an IMF. At this point, $u_{k}\left(t\right)$ is the first IMF component $C_{1}\left(t\right)$, representing the highest frequency component of the signal. The residue $r_{1}(t)$ is subsequently obtained as follows:
  (3) $$r_{1}(t)=x\left(t\right)-C_{1}\left(t\right)$$
(4) Each IMF encompasses different frequency components of the signal, ranging from high to low frequencies, representing the inherent modal characteristics of nonlinear signals, which vary with the signal’s evolution. By setting *x*(*t*) to $r_{n}(t)$, the aforementioned process is reiterated until the final residue $r_{n}(t)$ remains constant or monotonic, with *N* representing the number of decomposition layers. Consequently, *x*(*t*) is decomposed into
  (4) $$x(t)=\sum_{i=1}^{N}C_{i}(t)+r_{n}(t)$$

### 3.2. Complementary Ensemble Empirical Mode Decomposition

The Complementary Ensemble Empirical Mode Decomposition (CEEMD) is an improved method proposed to overcome the mode mixing problem in the EMD algorithm, as well as the shortcomings of EEMD being affected by low-frequency noise. The algorithm process is as follows [17]: First, white noise is added to the original signal to generate the first IMF with noise. Then, the decomposition process is repeated for the signal, using different noise sequences each time, and finally, a more accurate first IMF is obtained through ensemble averaging. Next, this more accurate first IMF is taken as the new signal for decomposition, and the ensemble average is calculated to obtain the second IMF component. This process is repeated to obtain the *N*-th IMF component, and finally, the original signal is decomposed into

(5) $$C_{1}(t)=\frac{1}{N}\sum_{i=1}^{N}E_{1}\left[x(t)+\delta w_{i}(t)\right]$$

Here, $w_{i}\left(t\right)$ represents the Gaussian white noise added to the original signal at the *i*-th layer, and $E_{i}$[] denotes the generation of the *i*-th IMF component. Then, $r_{1}(t)+\delta E_{1}[w_{i}(t)](i=1,2,\ldots ,N)$ is set as the new signal for decomposition. When the first IMF component is obtained, it is necessary to calculate the ensemble average, which is the second IMF component $C_{2}\left(t\right)$, given by

(6) $$C_{2}(t)=\frac{1}{N}\sum_{i=1}^{N}E_{1}\left[r_{1}(t)+\delta E_{1}\left[w_{i}(t)\right]\right]$$

Repeating the aforementioned steps yields the (*n* + 1)-th IMF component $C_{n+1}\left(t\right)$. In Equation (6), δ represents a proportionality constant, which is given by

(7) $$C_{n+1}(t)=\frac{1}{N}\sum_{i=1}^{N}E_{1}\left[r_{n}(t)+\delta E_{n}\left[w_{i}(t)\right]\right]$$

(8) $$x(t)=\sum_{i=1}^{N}C_{i}(t)+R(t)$$

The value of the proportional constant delta in CEEMD is crucial, generally in the range of 0.1–0.5. It determines the amplitude of the added white noise, which in turn affects the decomposition effect. Based on the published related work and comparative experiments, when delta is 0.2, the error between the reconstructed signal and the original signal is the smallest. Therefore, in this study, the value of delta is 0.2. Regarding the number of ensemble averaging times, based on past relevant research experience and the analysis of the characteristics of micro-grid load data, we set it to the default value of 50.

## 4. CEEMD-MIWOA-LSTM Microgrid Power Load Short-Term Forecasting Model

The LSTM network possesses numerous parameters, which significantly influence the efficacy and performance of the forecasting model. Consequently, this study employs the Multi-Strategy Improved Whale Optimization Algorithm (MIWOA) to optimize the parameters of the LSTM prediction model. The WOA is a metaheuristic optimization algorithm inspired by the hunting and attacking strategies of humpback whales. Compared to other swarm optimization algorithms, WOA simulates predatory behavior using random or optimal search agents and employs spirals to mimic the bubble-net hunting strategy of humpback whales [18].

### 4.1. Whale Optimization Algorithm

The WOA consists of three core stages: encircling, bubble-net attacking, and prey searching. In WOA, the position of each humpback whale corresponds to a potential solution. During the optimization process, the whales dynamically update their position vectors to gradually converge towards the global optimum in a multidimensional space. The algorithm initially contracts the search space through the encircling phase, followed by simulating the interaction of random and spiral movements in the bubble-net attacking phase to enhance the balance between local and global exploration of the solution space. Finally, WOA further optimizes global exploration during the searching phase to avoid getting trapped in local optima. The detailed steps of the WOA are as follows:

(1) Searching and Encircling Prey

In the WOA, individual whales navigate through the global solution space via random exploration to pinpoint the optimal solution. Given the unknown exact location of the optimal solution, WOA employs the best candidate solution from the current iteration as an approximate target, treating this solution as the target prey in the optimization process. During each iteration, all candidate solutions update their positions based on the current best solution, thereby contracting the search area and progressively approaching the optimal solution. This dynamic updating mechanism effectively enhances the algorithm’s convergence rate and solution accuracy by steering candidate solutions towards the current best position.

(9) $$\vec{D}=|\vec{C}\cdot {\vec{X}}^{*}(t)-\vec{X}(t)|$$

(10) $$\vec{X}(t+1)={\vec{X}}_{\mathrm{rand}}-\vec{A}\cdot \vec{D}$$

In the aforementioned formula, *t* represents the current iteration number. ${\vec{X}}^{*}$ denotes the best individual position of the whales. $\vec{A}$ and $\vec{D}$ are coefficient vectors, as detailed below:

(11) $$\vec{A}=2\vec{a}\cdot \vec{r}-\vec{a}$$

(12) $$\vec{c}=2\vec{r}$$

In the given context, the value of $\vec{a}$ linearly decreases within the range [0, 2] as the number of iterations increases. The value of $\vec{r}$ is a random number within the range [0, 1]; hence, $\vec{A}$ is a random number within the range [−1, 1].

(2) Bubble-net Feeding

As depicted in Equation (13), when humpback whales simulate bubble-net feeding behavior, and the position update between the whales and their prey is described by a logarithmic spiral equation. This spiral model effectively emulates the motion trajectory of humpback whales approaching their prey along a gradually contracting path, thereby facilitating the approximation of the prey.

(13) $${\vec{X}}^{\left(t+1\right)}=\vec{D}\cdot e^{bl}\cdot \cos(2\pi t)+{\vec{X}}^{*}(t)$$

In Equation (13), ${\vec{D}}^{\prime }$ represents the distance from the *i*-th whale to the target prey, b is an internal parameter controlling the degree of spiral contraction, and l is a random variable within the range [−1, 1] that simulates the random behavior of humpback whales during the predation process. To realistically simulate the bubble-net feeding behavior, the WOA assumes that each randomly selected whale has a 50% probability of moving along a circular path and a 50% probability of moving along a spiral path during the feeding process. Specifically, the circular path corresponds to the whales’ direct linear strategy of moving towards the target prey, while the spiral path utilizes a logarithmic spiral equation to describe the complex trajectory of whales gradually closing in on their prey:

(14) $${\vec{X}}^{\left(t+1\right)}=\left\{\begin{array}{ll} {\vec{X}}^{*}(t)-\vec{A}\cdot \vec{D}, & \mathrm{if}\;p\leq 0.5\\ \vec{D}\cdot e^{bl}\cdot \cos(2\pi t)+{\vec{X}}^{*}(t), & \mathrm{if}\;p\geq 0.5 \end{array}\right.$$

*p* is a random number within the range [0, 1].

(3) Prey Searching

To ensure that the whale population can thoroughly search the solution space, the WOA randomly selects an individual from the current population as the global reference optimal solution in each iteration. Other whale individuals then update their positions around this optimal solution, thereby accelerating convergence to the global optimum.

(15) $$\vec{D}=|\vec{C}\cdot {\vec{X}}_{\mathrm{rand}}-\vec{X}|$$

(16) $$\vec{X}(t+1)={\vec{X}}_{\mathrm{rand}}-\vec{A}\cdot \vec{D}$$

During the initialization phase of the algorithm, each whale starts from a random position within the solution space. Subsequently, in each iteration, each individual updates its position vector based on the best solution obtained in the current iteration or based on the position of a randomly selected individual. This updating mechanism not only enhances the diversity of the population but also reduces the likelihood of the algorithm becoming trapped in local optima. As the iterative process progresses, the best solution is continuously updated, gradually approaching the global optimum.

### 4.2. Multi-Strategy Improved Whale Optimization Algorithm

#### 4.2.1. Population Initialization

Compared to traditional metaheuristic algorithms, the WOA exhibits superior performance but still suffers from poor population diversity and a tendency to fall into local optima. To enhance convergence performance, the following three improvement methods are employed to refine the whale search algorithm:

1. Population Initialization Based on Tent Map

In response to the issue of poor initial population diversity in the WOA, this study employs the Tent chaotic map to replace the original method for population initialization. The Tent chaotic map, known for generating sequences with high randomness and uniform distribution, contributes to enhancing population diversity [19]. The specific formula is presented as follows:

(17) $$x_{n+1}=\left\{\begin{array}{c} 2x_{n},x_{n}\in [0,0.5)\\ 2(1-x_{n}),x_{n}\in [0.5,1) \end{array}\right.$$

**Figure 1:**
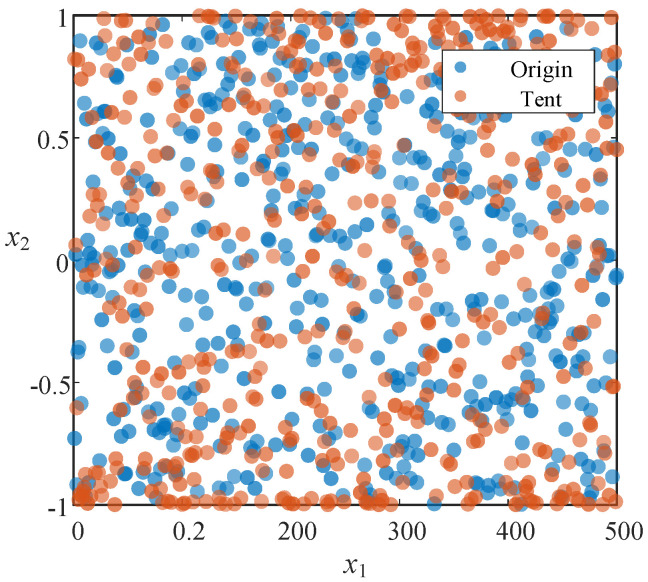
Population initialization comparison.

Let xn and xn + 1 represent the population positions at the *n*-th and (n + 1)-th generations, respectively. Figure 1 illustrates the initial populations generated by the two population initialization methods. Compared to the traditional initialization method, the Tent map generates a population that exhibits greater randomness and convenience, effectively enhancing the diversity of the initial population and providing the algorithm with improved global search capabilities.

2. Sinusoidal Chaotic Mapping

In Equations (11) and (12), the value of $\vec{a}$ linearly decreases within the range [0, 2] as the number of iterations increases, and $\vec{r}$ is a random number within the range [0, 1]. To enhance the randomness of the parameters and enable escape from local optima during position updates, this study proposes the use of Sinusoidal Chaotic Mapping to update $\vec{a}$. The principle of Sinusoidal Chaotic Mapping is as follows:

(18) $$x_{i+1}=ax_{i}^{2}\sin(\pi x_{i})$$

**Figure 2:**
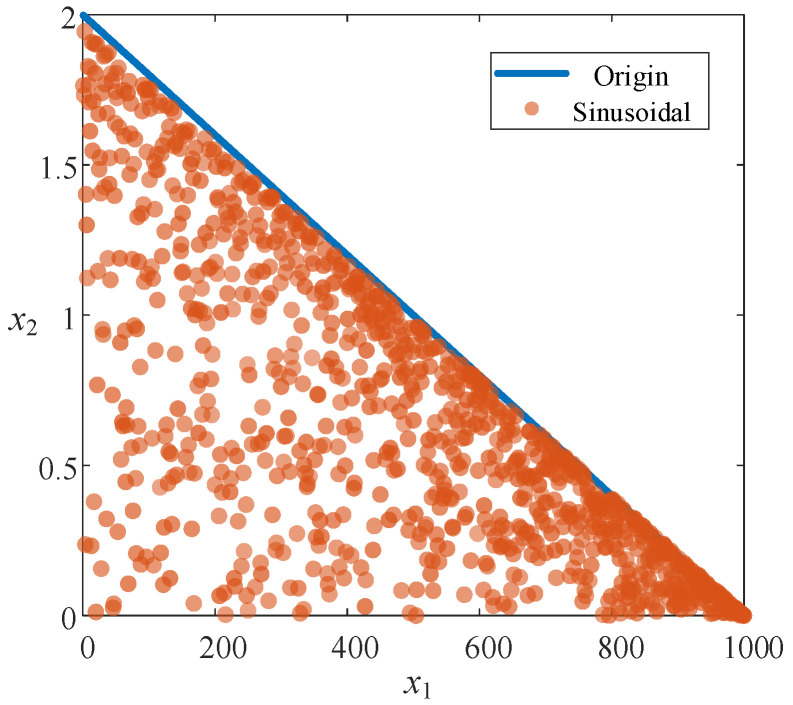
Sinusoidal Chaotic Mapping comparison.

In which *a* is the control parameter, typically set to 2.3. The chaotic orbit state values range from (0, 1). As shown in Figure 2, $\vec{a}$, which is in Equation (11), is uniformly distributed within the range (0, 2).

3. Lagrange Interpolation Method

To further enhance the local search capability and convergence speed of the WOA, the Lagrange interpolation method is introduced to simulate polynomials [20]. The expression for Lagrange interpolation is

(19) $$L_{n}(x)=\sum_{i=0}^{n}f_{i}l_{i}(x)$$

In the equation, *l_i_*(*x*) denotes the Lagrange polynomial, the expression of which is given by li(x)=∏j=0,j≠inx−xjxi−xj, and *f_i_* represents the value of the objective function. Three points from the *j*-th dimension of the global best solution *gbest* are selected for information generation, and Lagrange interpolation is performed on them, with one being the current global best solution and the remaining two being perturbations near the best solution. The expression for the aforementioned relationship is

(20) $$\sigma =rand\eta \cdot v(i,j)\;x_{0}(j)=gbest(j)\;x_{1}(j)=gbest(j)+\sigma \;x_{2}(j)=gbest(j)+\sigma$$

In the equation, η=0.5n denotes the population size, and v(i,j) represents the optimal velocity of the bat at each iteration according to the objective function. *x*_0_, *x*_1_, *x*_2_ in the *j*-th dimensional space can generate a parabolic curve through Lagrange interpolation, from which the minimum value can be obtained. The objective function value of the current best solution is compared with the minimum value obtained from the above process to update the position information. The computational expression for Lagrange interpolation is as follows:

(21) $$\begin{array}{ll} f(x)= & y_{0}\frac{\left(x-x_{1})(x-x_{2}\right)}{\left(x_{0}-x_{1})(x_{0}-x_{2}\right)}+y_{1}\frac{\left(x-x_{1})(x-x_{2}\right)}{\left(x_{1}-x_{0})(x_{1}-x_{2}\right)}\\ & +y_{2}\frac{\left(x-x_{0})(x-x_{0}\right)}{\left(x_{2}-x_{0})(x_{2}-x_{1}\right)} \end{array}$$

#### 4.2.2. Multi-Strategy Improved WOA Model

Chaotic initialization based on the Tent map generates an initial population with high randomness and uniform distribution, providing a broader search starting point for the algorithm, increasing population diversity, and enabling the algorithm to explore more potential solution spaces in the initial stage, thus reducing the possibility of falling into local optima. The sine chaotic map is used to update parameters, enhancing the randomness of parameters and enabling the algorithm to escape local optimal traps and continue searching for better solutions during the search process. The Lagrange interpolation method improves the algorithm’s local search capability by updating positions, enabling more precise searching for optimal solutions within a local range and accelerating the algorithm’s convergence speed.

The specific optimization process of MIWOA is depicted in Algorithm 1. Additionally, to validate the optimization performance of MIWOA, a comparative test is conducted using six benchmark test functions from CEC2005 against four other metaheuristic algorithms.
**Algorithm 1** MIWOA pseudo-code
**Input**: whale population *P_w_*
**Output:** best whale optimal position *P_bs_*1:Tent Mapping Initialize population size *N*, dimension *D*, the maximum number of iterations *T_max_*;2:Sinusoidal Chaotic Mapping Initialize *f_obj_*, *a*, *A*, *C*;3:Randomly generate N individuals in the space by Henon;4:Calculate the population fitness and find the optimal individual;5:**while** t ≤ *T_max_* **do**6:        Calculate new whale position *D_s_*7:        **for** *i* = 1 **to** *N* **do**8:        Compute *a*, A, *C*, *l*;9:            **if**
*p* < 0.5 **then**10:                **if**
*|A|* < 1 **then**11:                    execute shrink-wrap strategy12:
                **else**
13:                execute prey-search strategies14:
                **end if**
15:
            **else**
16:                execute spiral-update strategy17:
            **end if**

Lagrange Interpolation Method for Position Update18:
        **end for**
19:        *t* = *t* + 120:**end while**21:Return *P_bs_*.

#### 4.2.3. Verification of the MIWOA Optimization Model

This paper employs six CEC2005 benchmark test functions (F2, F4, F6, F9, F14, F22) to validate algorithm performance. The benchmark test functions include unimodal test functions, multimodal test functions, and fixed dimensional multimodal functions, aimed at evaluating from the perspectives of local and global optimization. The benchmark test functions are detailed in Appendix A. F2 and F4 are unimodal functions. F2 is a high condition unimodal function with rotational transformation, and the curved surface presents a multi-ridge unimodal structure. The bottom curve is the contour line of the function; the closer it is to the global optimum, the denser the contour lines, and the smaller the function value. F4 is a unimodal quadratic function with superimposed noise, and the curved surface is a smooth unimodal funnel structure. The closer the contour lines at the bottom are to the global optimum, the smaller the function value. F6, F9, F14, and F22 are multimodal functions. F6 is a multimodal function with superimposed Gaussian noise. The curved surface is not smooth and continuous but has random fluctuations, which simulates the interference in practical optimization problems. The global optimum is located at the lowest point of a certain valley. F9 is a multimodal function with high-frequency fluctuations (Rastrigin function). There are a large number of local peaks/valleys in the curved surface, and the global optimum is hidden among many local optima. The contour lines correspond to the high-frequency fluctuations of the surface, and the algorithm finds the global optimum among a large number of local optima. F14 is a multimodal composite function with discontinuous platforms, and the curved surface is divided into two parts, including the yellow platform area above (invalid search area) and the dense multimodal valley area below (including the global optimum). The vertical mutation boundary between the platform and the valley area simulates discontinuous optimization scenarios. The algorithm needs to first cross the platform boundary and then find the global optimum in the multi peak valley area. The F22 is a rotational mixed composite function with a high condition number matrix. The central region of the curved surface presents a “funnel-shaped” rapid descent, while the peripheral region is a flat plateau/multimodal structure. The six functions cover a variety of complex scenarios, testing the algorithm’s ability to balance global exploration and local development while also challenging its ability to deal with noise interference and spatial distortion problems.

**Figure 3:**
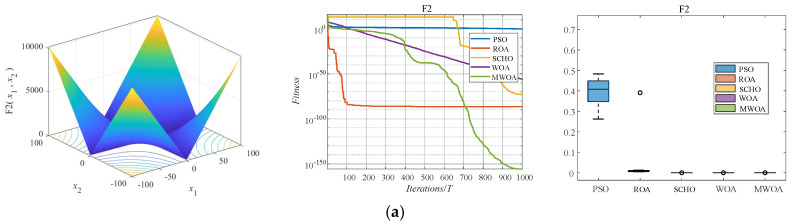

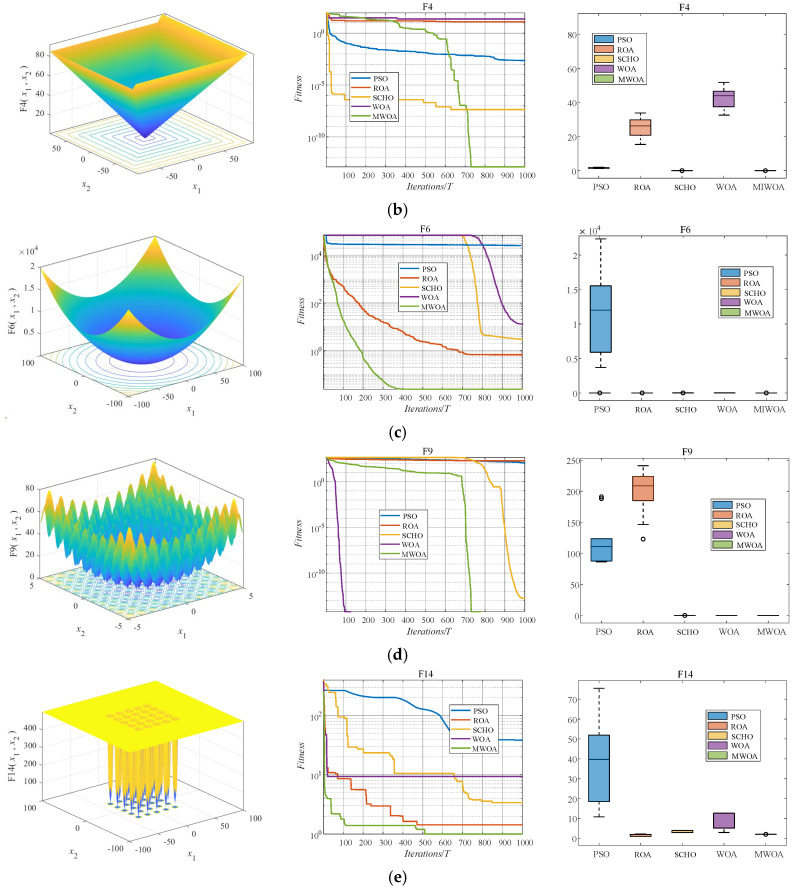

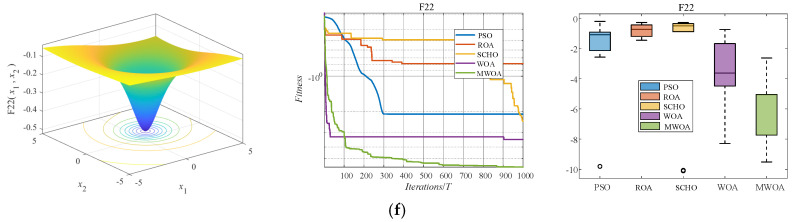
Comparison of benchmark function algorithm performance. (**a**) Test function F2, the convergence curves and error distributions after running 30 times. (**b**) Test function F4, the convergence curves and error distributions after running 30 times. (**c**) Test function F6, the convergence curves and error distributions after running 30 times. (**d**) Test function F9, the convergence curves and error distributions after running 30 times. (**e**) Test function F14, the convergence curves and error distributions after running 30 times. (**f**) Test function F22, the convergence curves and error distributions after running 30 times.

The paper selects five algorithms, namely Particle Swarm Optimization (PSO), Rodent Swarm Optimization (ROA), Sparrow Search Optimization (SCHO), Whale Optimization Algorithm (WOA), and MIWOA, to compare and verify the advantages of MIWOA. The convergence speed and accuracy of MIWOA, as well as the robustness of the algorithm, are evaluated through convergence curves and boxplots. Each function is independently executed 30 times. The test results on CEC-2005 are illustrated in Figure 3 and Table 1 and Table 2.

Analyzing Table 2 and Table 3, where “Best” represents the optimal value, “Mean” indicates the average value, and “Std” denotes the standard deviation, reveals that MIWOA consistently achieves superior optimization metrics across both unimodal and multimodal test functions. Over the course of 30 trials, MIWOA demonstrates rapid and stable convergence, particularly in benchmark functions F2 and F4, outperforming the standard WOA. In the case of benchmark function F6, MIWOA successfully avoids local optima and converges rapidly. For functions F9 and F14, while all other comparative algorithms are trapped in local optima, MIWOA effectively escapes these traps and converges. Similarly, in F22, MIWOA reaches the optimal value. The superior performance of MIWOA is largely attributable to its Tent initialization, parameter updates, and position updating through Lagrange interpolation, enabling both rapid convergence to optimal solutions and avoidance of local optima. By comparing the running times over 30 runs, it can be seen that the proposed method increases the running time, thus raising the complexity. However, as can be observed from multiple result graphs in Figure 3, it effectively improves the convergence speed and convergence accuracy. The increase in complexity is within an acceptable range. Compared to traditional algorithms, MIWOA’s three-fold improvement approach significantly enhances both convergence speed and optimization accuracy. Overall, MIWOA exhibits superior optimization performance relative to other metaheuristic algorithms.

### 4.3. MIWOA-LSTM Method

(1) Fundamental Principles of LSTM

**Figure 4:**
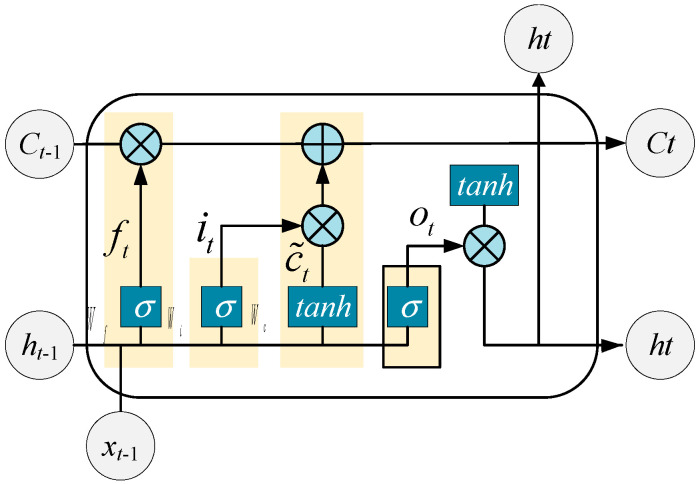
LSTM structure diagram.

Hochreiter et al. introduced a novel recurrent network architecture known as the Long Short-Term Memory neural network [21]. The LSTM enhances long-term memory capability by incorporating three types of gates—forget gate, input gate, and output gate—that serve as control units to manage the cell state updates. This design effectively addresses the issues of gradient vanishing and gradient explosion commonly encountered in traditional RNNs [22]. LSTM has significant technological advantages. It has a unique gating mechanism that can accurately capture long-term dependencies and short-term mutation features in power sequences, effectively circumventing the gradient vanishing and exploding problems that afflict traditional Recurrent Neural Networks (RNNs), and thus achieves superior convergence efficiency. In microgrid power forecasting, this feature significantly improves the accuracy of power load forecasting, reduces error fluctuations, and has strong nonlinear fitting ability, which can adapt to the randomness and intermittency of distributed power generation output, thereby significantly improving the accuracy of power load forecasting. The structure of an LSTM unit is illustrated in Figure 4.

The inputs to the LSTM at the current time step *t* include the input sequence $x_{t}$, the cell state from the previous time step $C_{t-1}$, and the hidden state from the previous time step $h_{t-1}$. The outputs generated at time t are the current cell state *C_t_* and the current hidden state $h_{t}$. The specific calculations are as follows:

(22) $$\left.f_{t}=\sigma \left(W_{f}\cdot \left[h_{t-1},x_{t}\right.\right]+b_{f}\right)$$

(23) $$i_{t}=\sigma (W_{i}\cdot [h_{t-1},x_{t}]+b_{i})$$

(24) $$o_{t}=\sigma (W_{o}\cdot [h_{t-1},x_{t}]+b_{o})$$

(25) $${\tilde{c}}_{t}=tanh(w_{c}\cdot \left[h_{t-1},x_{t}\right]+b_{c})$$

(26) $$c_{t}=f_{t}⊙c_{t-1}+i_{t}⊙{\tilde{c}}_{t}$$

(27) $$h_{t}=o_{t}⊙tanh(c_{t})$$

In these equations, *f_t_*, *i_t_*, and *o_t_* represent the computed states of the forget gate, input gate, and output gate at the current time step *t*; *W_f_*, *W_i_*, and *W_o_* are the weight matrices for the forget, input, and output gates, respectively; *b_f_*, *b_i_*, *b_o_* denote the bias terms associated with these gates; ${\tilde{c}}_{t}$ is the cell state at the current time step *t*; *W_c_* is the weight matrix for the cell state; *c_t_* represents the output at time *t*; *b_c_* is the cell state bias term; σ denotes the sigmoid activation function; tanh is the hyperbolic tangent activation function; and ⊙ represents element-wise multiplication.

(2) Optimization of Key Parameters in LSTM

The maximum number of iterations, initial learning rate, and the number of neurons in the hidden layers are critical parameters that significantly affect the training and prediction accuracy of LSTM models. Traditional methods of parameter selection based on manual tuning are subjective and often undermine the predictive accuracy of LSTM models. To enhance model precision, this study proposes the use of MIWOA for optimizing LSTM’s key parameters. The MIWOA optimization process for LSTM is as follows:

(1) Data Preparation: Load and preprocess power generation data, then construct the initial LSTM model. Parameter setting as follows:
  Input nodes: 96; Onput nodes: 96; Hidden layers: 2; Learning rate: [0.01, 0.8].

(2) Parameter Initialization: Initialize the population parameters and define the search range for LSTM’s parameters.
(3) Fitness Evaluation: Determine the fitness value within the MIWOA to identify the optimal individual position.
(4) Optimization Using MIWOA Enhancements: Improve MIWOA through Tent chaotic mapping for population initialization, Sinusoidal Chaotic Mapping, and Lagrange interpolation, updating positions to obtain the optimal parameters for LSTM.
(5) Model Optimization: Substitute the optimal parameters into the LSTM model to create the optimized LSTM configuration.

### 4.4. MIWOA-LSTM

Incorporating the MIWOA search algorithm, CEEMD technology, and LSTM model, a hybrid forecasting model is constructed. CEEMD decomposes the original load time series into multiple IMFs and a residual component. Each IMF represents components with different frequencies and characteristics in the original data, enabling a clear separation of high-frequency short-term fluctuations from low-frequency long-term trends. Subsequent prediction models can then perform more accurate modeling for components with distinct characteristics, thereby improving the overall prediction accuracy. MIWOA enhances its search capabilities through multiple strategy improvements such as chaotic initialization, sine-chaotic mapping, and Lagrange interpolation. This enables it to rapidly identify better parameter solutions within the vast parameter space, facilitating the LSTM model to converge more quickly during the training process and more accurately capture the changing trends of microgrid loads during prediction. The hidden layer of LSTM can learn complex feature representations in the data. These feature representations play a crucial role in the prediction process. LSTM has a powerful ability to handle long- and short-term dependencies in time–series data. When dealing with microgrid load data, it can make full use of the useful information in historical data to accurately predict future load changes. The process of this model is as follows:

(1) Data Preprocessing: Preprocess the power load data of microgrid buildings.
(2) Population Initialization: Generate an initial population with high randomness and uniform distribution according to the Tent mapping Formula (17).
(3) Parameter Initialization: Initialize the key parameters such as $\vec{a},\vec{A},\vec{C}$ in the algorithm using Equations (9)–(16).
(4) Fitness Evaluation: Apply the initialized population to the LSTM model, train it on the training dataset, calculate the fitness values of the population, and select the best individual in the initial population.
(5) Position Update: Randomly generate a number *p* within the range of [0, 1] in each iteration to determine the update strategy.
(6) Contraction–Encirclement: When *p* < 0.5 and $|\vec{A}|<1$, execute the contraction–encirclement strategy. Update the position through Equations (9)–(12) to accelerate convergence.
(7) Prey Search: When *p* < 0.5 and $|\vec{A}|>1$, execute the prey search strategy. Update the position through Equations (15) and (16) to increase diversity.
(8) Spiral Update: When *p* > 0.5, execute the spiral update strategy. Simulate the spiral movement trajectory of whales during predation through Equations (13) and (14).
(9) Local Optimization: After each position update, use the Lagrange interpolation method (19–20) to perform local optimization on the position, improving the local search ability and convergence speed of the algorithm.
(10) Model Construction: When *T*_max_ is reached, the algorithm stops running. At this time, the parameter combination of the LSTM model represented by the current optimal whale individual is the optimal parameter, which is used for the short-term power load forecasting of microgrid buildings.

## 5. Load Forecasting Analysis for Microgrid Buildings Based on CEEMD-MIWOA-LSTM

### 5.1. Microgrid Load Dataset

**Figure 5:**
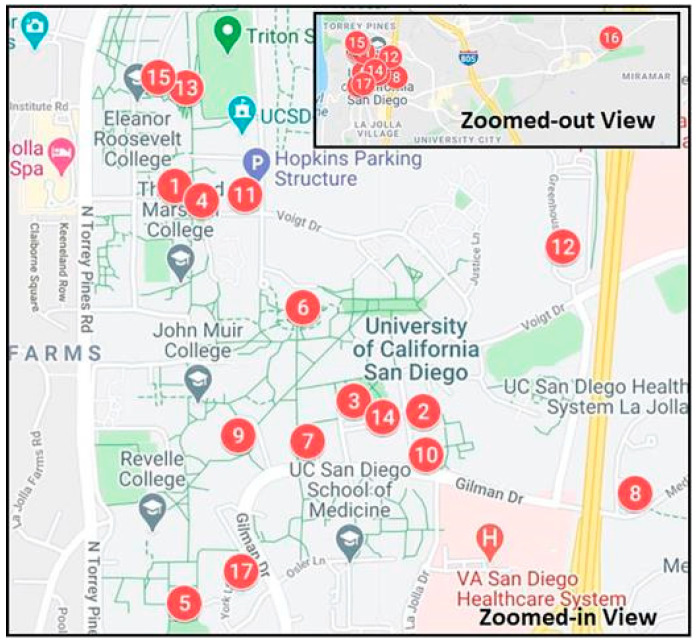
Microgrid system at the University of California, San Diego (UCSD) [22].

To validate the methodology proposed in this study, the UCSD dataset was selected for real-world application. The UCSD Microgrid Dataset, collected from the microgrid system at the University of California, San Diego (UCSD), is one of the advanced campus-level microgrids in North America and is recognized as one of the world’s leading microgrids, encompassing a variety of energy generation, storage, and consumption devices. UC San Diego hosts a central natural-gas-fired plant with two high efficiency 13.5 MW combined cycle co-generation Solar Turbines Titan 130 turbines and a 3 MW Dresser-Rand steam turbine, 10 million gallons of chilled thermal energy storage, 3 MW distributed solar PV generators, a 2.8 MW fuel cell that is the largest on a US college campus, 2.5 MW battery energy storage systems, 125 electric vehicle charging stations (many with dual ports), and energy efficient campus buildings with controllable loads [23]. In this research, the authors have chosen microgrid load prediction data from the following four buildings: Center Hall, Police Building, Music Building, and Student Services. It is noteworthy that these four buildings exhibit distinct typical data characteristics based on their different uses, as shown in Figure 5.

Location of buildings and facilities: (1) Robinson Hall, (2) Pepper Canyon Hall, (3) Student Services Center, (4) Social Sciences, (5) Galbraith Hall, (6) Geisel Library, (7) Center Hall, (8) East Campus Office, (9) Mandeville Center, (10) Gilman Parking, (11) Hopkins Parking, (12) Police Department, (13) Otterson Hall, (14) Music Building, (15) Rady Hall, (16) Trade Street Warehouse, and (17) Central Utility Plant.

### 5.2. CEEMD of Load Time-Series Data

**Figure 6:**
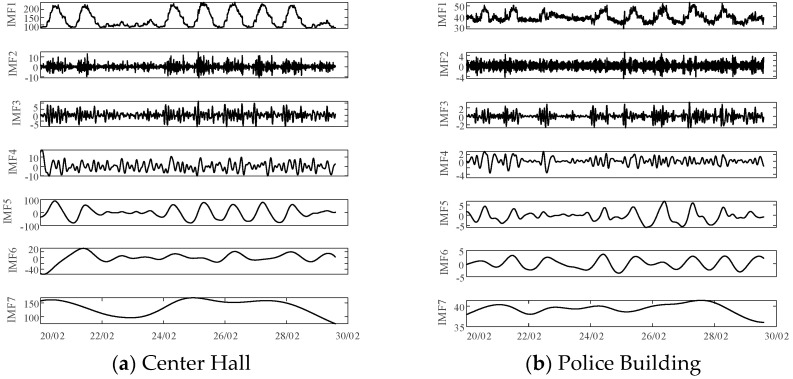

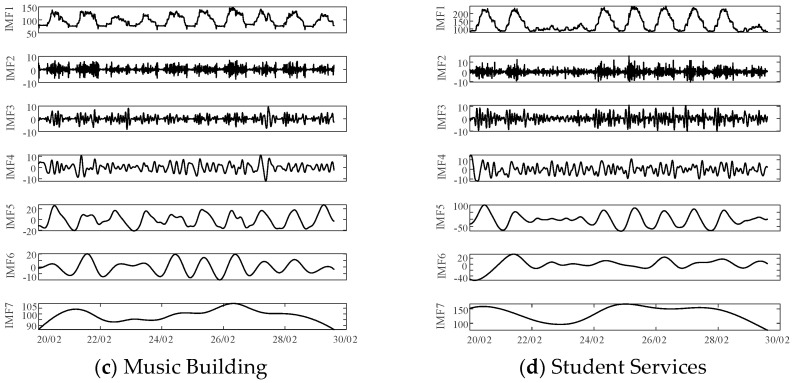
Decomposition of ten-day load time series.

The load data for the period from 20 February to 30 February, spanning a duration of 10 days, for the four buildings—Center Hall, Police Building, Music Building, and Student Services—was decomposed using CEEMD. The results, as depicted in Figure 6, demonstrate a reduction in noise within the high-frequency components and a more stable signal trend.

### 5.3. Analysis of Forecasting Results

(a) Center Hall

**Figure 7:**
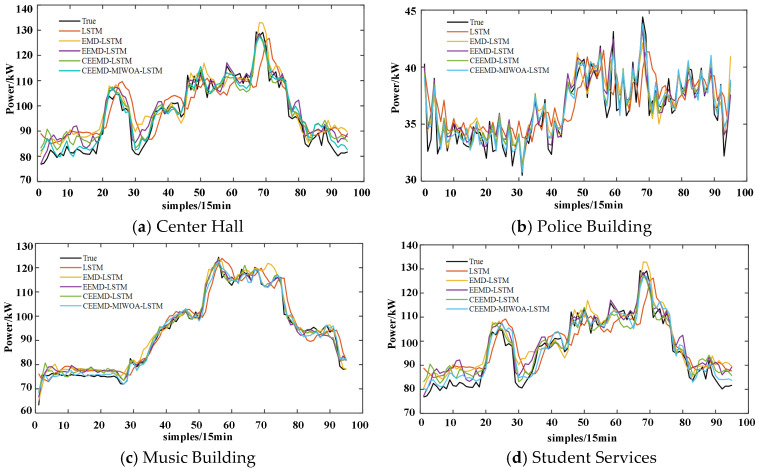

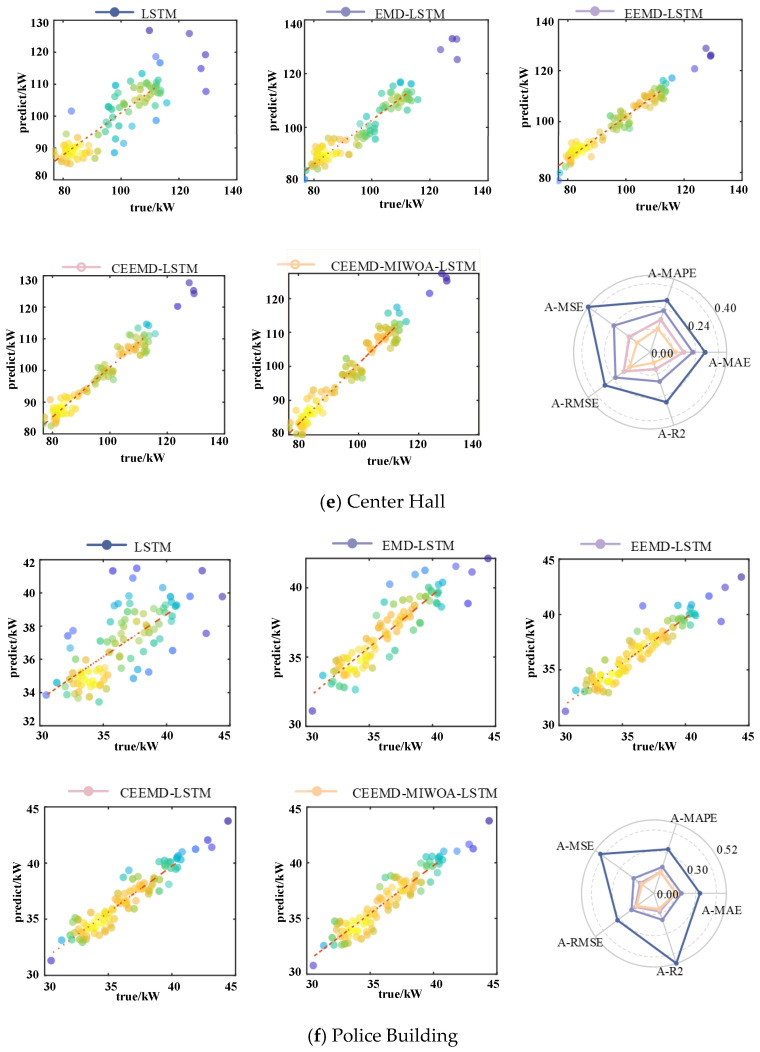

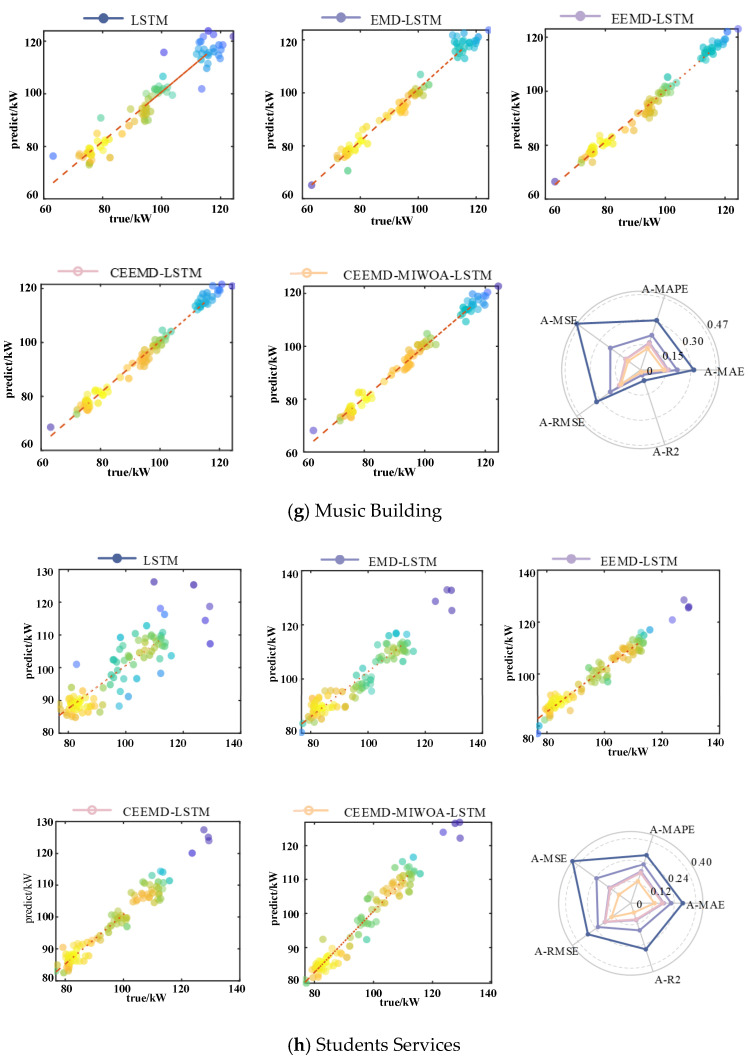
Load forecasting in microgrids for different buildings under various models.

The Center Hall, primarily utilized for teaching and conferences, exhibits higher electricity consumption during class hours, leading to potential periodic fluctuations. It can be observed that on 22, 23, and 29 February, which are rest days, the load is at a lower level, while on working days, the load fluctuates between day and night. In this study, several methods were compared, and the results are shown in Figure 7a,e. To evaluate the prediction performance of the model, we used multiple prediction error metrics, including MSE, RMSE, MAE, MAPE, and *R*^2^. Each of these metrics has its own advantages and disadvantages. For example, MSE is sensitive to outliers, while MAE gives equal weight to all errors. MAPE represents the relative error between the predicted value and the true value in percentage form and pays more attention to the relative error. A detailed discussion and application of these metrics can be found in the relevant literature [3]. From the figure, it can be discerned that for short-term forecasting of the Center Hall, the method proposed in this study demonstrates the best predictive accuracy, effectively tracking the load variations.

(b) Police Building

The Police Building, as a campus security facility, typically maintains a relatively stable pattern of electricity consumption but may have special load demands during nights or when incidents occur. The data reveals higher loads from the afternoon into the night and, compared to other buildings, it maintains a higher load even on rest days. A comparison of different methods for load forecasting is depicted in Figure 7b,f.

(c) Music Building

The Music Building, akin to a conservatory or performing arts venue, may experience increased electricity usage during events or performances. For such structures, load forecasting may need to consider external factors such as event schedules. The data indicates higher loads during the day and, compared to other buildings, a consistent load at all times. A comparison of different methods for load forecasting is shown in Figure 7c,g.

(d) Student Services

The Student Services building, a public facility typically operational throughout the day, may also experience increased load during peak daytime hours, with trends and load volumes similar to the Center Hall. A comparison of different methods for load forecasting is presented in Figure 7d,h.

The CEEMD-MIWOA-LSTM model, targeting the different load characteristics of Center Hall, Police Building, Music Building, and Student Services, achieves precise tracking of load variations in each building through CEEMD and MIWOA-optimized LSTM model parameters. Meanwhile, the paper also includes comparative analysis of models such as LSTM, EMD-LSTM, and EEMD-LSTM, highlighting the advantage of this model in adapting to different load curves with higher prediction accuracy and lower error.

In microgrids, the load characteristics of different buildings exhibit distinct features. Through the analysis of short-term load forecasting results presented in Figure 7 and Table 3, it is observed that the EMD-LSTM model, which incorporates signal processing, outperforms the model that solely utilizes LSTM. The CEEMD-MIWOA-LSTM model proposed in this study demonstrates the best performance. After optimizing the original parameters of the model for different datasets, it adapts to various types of load changes. For the loads of Center Hall and Police Building, which are more volatile and irregular, the R^2^ values reached 0.95934 and 0.9332, respectively. In the case of Music Building, where the load fluctuations are more regular, the R^2^ value achieved 0.99043, enabling a near-perfect prediction of future short-term loads.

**Table 3:** Comparative results of different models.

Building	Predict Model	MSE	RMSE (kw)	MAE (kw)	MAPE	R^2^
Center Hall	LSTM	50.3184	7.0935	5.7571	0.06153	0.72498
	EMD-LSTM	29.5281	5.4340	4.4453	0.04933	0.83861
	EEMD-LSTM	16.9555	4.1177	3.4980	0.03876	0.90733
	CEEMD-LSTM	13.9457	3.7344	3.1399	0.03488	0.92234
	CEEMD-MIWOA-LSTM	**7.30200**	**2.7022**	**2.2392**	**0.02417**	**0.95934**
Police Building	LSTM	4.47520	2.1155	1.6729	0.04653	0.47880
	EMD-LSTM	1.69391	1.3015	0.9964	0.02766	0.80272
	EEMD-LSTM	1.16190	1.0779	0.7958	0.02231	0.86468
	CEEMD-LSTM	0.62332	0.7895	0.5935	0.01676	0.92224
	CEEMD-MIWOA-LSTM	**0.53549**	**0.7318**	**0.5579**	**0.01554**	**0.93320**
Music Building	LSTM	17.6593	4.2023	3.1878	0.03495	0.93308
	EMD-LSTM	8.36300	2.8919	2.1925	0.02444	0.96831
	EEMD-LSTM	4.10000	2.0248	1.6690	0.01929	0.98446
	CEEMD-LSTM	3.01580	1.7366	1.3613	0.01585	0.98849
	CEEMD-MIWOA-LSTM	**2.50780**	**1.5836**	**1.2551**	**0.01357**	**0.99043**
Student Services	LSTM	49.1501	7.0107	5.665	0.06031	0.73137
	EMD-LSTM	28.9090	5.3767	4.4017	0.04874	0.84200
	EEMD-LSTM	18.0506	4.2486	3.5999	0.03996	0.90134
	CEEMD-LSTM	13.8806	3.7257	3.1438	0.03474	0.92270
	CEEMD-MIWOA-LSTM	**7.56890**	**2.7512**	**2.2757**	**0.02422**	**0.95785**

## 6. Conclusions

To address the issues of high randomness and volatility in short-term load forecasting of microgrids, which lead to low prediction accuracy, this paper proposes a CEEMD-MIWOA-LSTM hybrid forecasting model. This composite model employs the CEEMD method for the decomposition and reconstruction of raw microgrid load data and effectively suppresses modal aliasing, making the decomposed components more stable and interpretable. The proposed MIWOA solves the defect of traditional optimization algorithms. By improving the WOA, MIWOA has enhanced its global search and local development capabilities. Through testing with the CEC2005 benchmark functions, the proposed MIWOA demonstrated superior convergence capabilities and precision, addressing the shortcomings of traditional optimization algorithms, such as slow convergence speed, poor population diversity, and susceptibility to local optima. After testing and analysis with four practical case studies, the determination coefficient (*R*^2^), Root Mean Square Error (RMSE), and mean absolute error (MAE) of the proposed method with advanced methods were compared. The results indicate that the proposed CEEMD-MIWOA-LSTM model achieved the highest R^2^ and the lowest RMSE and MAE in all four scenarios. For example, in a central hall scenario with significant fluctuations, the proposed model’s R^2^ (0.95934) increased by 3.8% compared to CEEMD-LSTM (0.92234), 5.4% compared to EEMD-LSTM (0.90733), and 12.5% compared to EMD-GRU (0.83861); in the music hall scene with load patterns, the R^2^ (0.99043) of the proposed model improved by 0.2% compared to CEEMD-LSTM (0.98849) and 0.6% compared to EEMD-LSTM (0.98446), indicating that the proposed method has significant advantages in prediction accuracy.

Although the model proposed in this paper has achieved good results in short-term load forecasting of micro-grids, in the future, more advanced optimization algorithms can be explored to reduce the time consumption and adapt to the dynamic characteristics. Secondly, in the future, the impact of extreme weather (such as strong storms, high temperatures) and emergencies (such as large-scale events in public buildings, equipment failures) on microgrid load can be considered to capture the laws of load changes. Finally, the interpretability of the model can be further explored.

## Figures and Tables

**Table 1:** Test the average of 30 fitness values when the maximum iteration count (*t* = 1000) is reached.

Algorithm	Metrics	F2	F4	F6	F9	F14	F22
PSO	Best	0.34	2.42 × 10^−2^	1.02 × 10^5^	1.42 × 10^1^	1.03 × 10^2^	−2.53
	Mean	3.81	2.66 × 10^−2^	1.92 × 10^5^	2.05 × 10^1^	1.18 × 10^2^	−1.07
	Std	2.27 × 10^−2^	1.01 × 10^−2^	1.42 × 10^5^	2.69 × 10^1^	1.24 × 10^2^	1.64
	Time (s)	136.9	121.3	160.6	214.3	180.8	220.4
ROA	Best	4.55 × 10^−18^	0.27	4.36 × 10^−1^	2.03 × 10^1^	2.33	−1.58
	Mean	3.48 × 10^−17^	1.84	9.33 × 10^−1^	2.33 × 10^1^	5.33	2.04 × 10^−1^
	Std	6.04 × 10^−17^	1.16	7.08 × 10^−1^	2.01 × 10^1^	4.70	2.62 × 10^−1^
	Time (s)	147.1	129.6	170.2	220.3	189.5	230.0
SCHO	Best	2.19 × 10^−73^	2.13 × 10^−7^	1.25 × 10^1^	2.04 × 10^−28^	2.04	−3.24
	Mean	3.06 × 10^−72^	2.43 × 10^−7^	1.63 × 10^1^	1.78 × 10^−28^	2.32	−2.18
	Std	3.46 × 10^−72^	2.03 × 10^−7^	1.13 × 10^1^	2.18 × 10^−28^	2.38	−3.12
	Time (s)	151.2	131.9	174.6	223.8	190.4	230.5
WOA	Best	1.06 × 10^−55^	1.17	9.65 × 10^−1^	0	1	−6.62
	Mean	2.88 × 10^−55^	1.43	1.14 × 10^1^	9.63 × 10^−120^	9.63	−3.31
	Std	4.22 × 10^−55^	1.21	1.62 × 10^1^	6.92 × 10^−110^	6.92	−5.29
	Time (s)	153.0	130.8	168.6	208.9	183.9	207.9
MIWOA	Best	**1.5 × 10** **^−^** **^153^**	**1.46 × 10** **^−^** **^38^**	**1.26 × 10** **^−^** **^1^**	**0**	**1**	**−** **9.48**
	Mean	**2.2 × 10** **^−^** **^153^**	**2.06 × 10** **^−^** **^38^**	**1.76 × 10** **^−^** **^1^**	**9.63 × 10** **^−^** **^220^**	**1**	**−** **5.84**
	Std	**1.1 × 10** **^−^** **^153^**	**1.06 × 10** **^−^** **^38^**	**1.53 × 10** **^−^** **^1^**	**1.82 × 10** **^−^** **^200^**	**1**	**−** **7.47**
	Time (s)	**171.4**	**146.6**	**179.9**	**224.0**	**197.5**	**219.0**

**Table 2:** Wilcoxon rank-sum test for test results.

	*p*-Value			
Algorithm	PSO	ROA	SCHO	WOA
F2	3.44 × 10^−4^	3.44 × 10^−4^	3.44 × 10^−4^	3.44 × 10^−4^
F4	1.83 × 10^−4^	1.83 × 10^−4^	1.83 × 10^−4^	1.83 × 10^−4^
F6	4.03 × 10^−4^	4.03 × 10^−4^	4.03 × 10^−4^	4.03 × 10^−4^
F9	6.01 × 10^−5^	6.01 × 10^−5^	6.01 × 10^−1^	6.48 × 10^−1^
F14	9.12 × 10^−5^	9.12 × 10^−5^	9.12 × 10^−5^	9.12 × 10^−5^
F22	4.22 × 10^−2^	1.65 × 10^−8^	1.65 × 10^−8^	2.08 × 10^−3^

## Data Availability

The original contributions presented in this study are included in the article. Further inquiries can be directed to the corresponding author.

## Appendix A. Benchmarking Function Formula

**Table:**

Benchmark Test Functions	Dim	Scale	Optimal
$f_{2}(x)=\sum_{i=1}^{n}\left|x_{i}\right|+\prod_{i=1}^{n}\left|x_{i}\right|$	30	[−10, 10]*^n^*	0
$f_{4}(x)=\underset{i}{\max}\{|x_{i}|,1\leq i\leq n\}$	30	[−100, 100]*^n^*	0
$f_{6}(x)=\sum_{i=1}^{n}{\left(\left|x_{i}+0.5\right|\right)}^{2}$	30	[−100, 100]*^n^*	0
$f_{9}(x)=\sum_{i=1}^{n}[x_{i}^{2}-10\cos(2\pi x_{i})+10)]$	30	[−5.12, 5.12]*^n^*	0
$f_{14}\left(x\right)={\left[\frac{1}{500}+\sum_{j=1}^{25}\frac{1}{j+\sum_{i=1}^{2}{\left(x_{i}-a_{ij}\right)}^{6}}\right]}^{-1}$	2	[−65.536, 65.536]*^n^*	0
$f_{22}(x)=-\sum_{i=1}^{7}{\left[(x-a_{i}){\left(x-a_{i}\right)}^{T}+c_{i}\right]}^{-1}$	4	[0, 10]*^n^*	−10
